# Factors influencing TSH suppression efficacy in postoperative papillary thyroid carcinoma patients: a retrospective cohort study

**DOI:** 10.1186/s12893-024-02426-y

**Published:** 2024-05-03

**Authors:** Qing Zhang, Zhen-Zhu Zhong, Tian Wu, Yuan-Qiang He

**Affiliations:** Thyroid and Breast Surgery Department, Ganzhou People’s Hospital, No.18 Meiguan Avenue, Zhanggong District, 341000 Ganzhou City, Jiangxi Province China

**Keywords:** Thyroid papillary carcinoma, Thyroid-stimulating hormone suppression therapy, Levothyroxine sodium, Hashimoto thyroiditis, Influencing factors

## Abstract

**Objectives:**

While surgery plays a crucial role in treating papillary thyroid carcinoma (PTC), the potential effects of subsequent TSH suppression therapy on prognosis should not be overlooked. This study aims to investigate the factors that influence postoperative TSH suppression therapy in patients with PTC.

**Methods:**

This study was a retrospective cohort study conducted at our hospital. It included 268 patients who underwent surgery and were pathologically diagnosed with PTC between February 2019 and February 2021. The selected patients received postoperative TSH suppression therapy. Based on the TSH level measured 12 months after surgery, the patients were divided into two groups: TSH level conforming group (*n* = 80) and non-conforming group (*n* = 188). We then compared the general clinical data, clinicopathological characteristics, preoperative laboratory test indicators, postoperative levothyroxine sodium tablet dosage, follow-up frequency, and thyroid function-related indicators between the two groups of patients. The correlation between the observed indicators and the success of TSH suppression therapy was further analyzed, leading to the identification of influencing factors for TSH suppression therapy.

**Results:**

There were no statistically significant differences in general clinical data and clinicopathological characteristics between the two groups of patients (*P* > 0.05). The proportion of patients with preoperative TSH ≥ 2.0 mU/L was higher in the non-conforming group compared to the TSH level conforming group (*P* < 0.05), and the ROC curve analysis indicated that the area under the curve for the preoperative TSH index was 0.610 (*P* < 0.05). The proportion of patients in the TSH level conforming group who took oral levothyroxine sodium tablets at a dose of ≥ 1.4 µg/kg·d after surgery was higher (*P* < 0.05). The postoperative levels of FT_3_ and FT_4_ were higher in the TSH level conforming group (*P* < 0.05). The results of binary logistic regression analysis indicated that factors “Postoperative TSH level ≥ 2 mU/L”, “Levothyroxine sodium tablet dose<1.4 µg/kg·d”, and “Combined with Hashimoto thyroiditis” were significantly associated with an elevated risk of postoperative TSH levels failing to reach the target (*P* < 0.05).

**Conclusion:**

Optimal thyroid function in patients with PTC post-surgery is best achieved when adjusting the dose of levothyroxine sodium in a timely manner to reach the target TSH level during follow-up visits.

## Introduction

Thyroid cancer (TC) is a prevalent malignant tumor found in the endocrine system and has shown a significant rise in incidence in the last thirty years [1]. TC can be divided into three distinct types based on their histomorphological characteristics, namely differentiated thyroid cancer (DTC), anaplastic thyroid cancer (ATC), and medullary thyroid cancer (MTC). DTC further includes two histological subtypes, papillary thyroid carcinoma (PTC) and follicular thyroid carcinoma (FTC). In recent years, there has been a growing number of TC diagnoses, with PTC being the most common type and demonstrating a notable upward trend [2, 3]. PTC accounts for approximately 80% of all TC [4]. It can manifest at any stage but is typically more prevalent in individuals aged thirty to fifty, with an average age of forty [5].

Thyroid-stimulating hormone (TSH), produced and released by the adenohypophysis, acts as a growth factor, triggering the synthesis and release of thyroid hormones [6]. Moreover, it enhances the growth of epithelial cells within the thyroid follicles. Nonetheless, the presence of thyroid hormones (T_4_) and triiodothyronine (T_3_) in the bloodstream can modulate the levels of TSH. When the levels of thyroid hormones and triiodothyronine increase, the TSH level will decrease in response. Research has revealed that the prolonged and excessive release of TSH leads to the stimulation of thyroid cell proliferation, thereby elevating the likelihood of developing TC [7]. At present, the principal approach to managing differentiated TC in our nation consists of surgery, followed by TSH suppression therapy and radioactive iodine treatment. For individuals diagnosed with PTC, the likelihood of tumor reappearance is categorized as either low-risk, intermediate-risk, or high-risk, relying on parameters like tumor dimensions, regional lymph node metastasis, vascular invasion, and molecular pathological traits. Different TSH suppression targets are set based on these risk stratifications [8].

TSH suppression therapy aims to lower serum TSH levels by gradually increasing the dosage of levothyroxine sodium tablets. Several factors, including gender, age, weight, preoperative TSH concentration, and diseases affecting drug absorption, can influence the required dosage [8–11]. The goal of TSH suppression therapy is to achieve low or undetectable TSH levels, leading to subclinical hyperthyroidism [12]. However, prolonged low TSH levels can have negative effects on the cardiovascular and skeletal systems [13, 14]. These adverse reactions not only impact patients’ quality of life but also outweigh the treatment benefits [15]. Therefore, individualized TSH suppression therapy plans should be formulated and regularly monitored. Close monitoring of thyroid hormone levels during medication is necessary to adjust the dosage timely and prevent the occurrence of hyperthyroidism or hypothyroidism, ensuring optimal therapeutic outcomes [16]. The suppression therapy of TSH is a crucial clinical strategy to hinder the relapse of PTC. A healthcare professional administers exogenous thyroid hormone to ensure that the level of TSH remains below the lower threshold of the normal range. Occasionally, patients may require significantly high oral doses of exogenous thyroid hormone to attain undetectable TSH levels. This approach not only provides the necessary thyroid hormone that patients with postoperative TC lack, but also inhibits the growth of DTC cells, effectively preventing the recurrence of PTC [17].

Although surgery plays a crucial role in treating PTC, it is important to consider the impact of successful TSH suppression therapy in achieving target thyroid-stimulating hormone levels on the overall prognosis. Surgery directly influences the patient’s treatment selection and follow-up and is closely linked to tumor prognosis. Nonetheless, restraining TSH therapy additionally assumes a substantial part in averting tumor reoccurrence. Consequently, it is of utmost importance to effectively administer postoperative TSH suppression therapy in the treatment of patients with PTC. In clinical practice, many patients receive either excessive or insufficient treatment after surgery, which exposes them to the risks of hyperthyroidism and hypothyroidism. To mitigate these side effects, it is essential to conduct risk assessments and closely monitor TSH target values during follow-up. This study aimed to compare observation indicators between patients with PTC who underwent thyroidectomy and achieved the standard TSH level after TSH suppression therapy, and those who did not reach the standard. The findings of this study can help in selecting appropriate treatment plans to improve treatment outcomes, enhance patients’ quality of life, and reduce medical expenses. Additionally, this research implies the significance of acknowledging the patients’ health utility value within the realm of nursing, potentially fostering future interdisciplinary cooperation between nursing and other fields.

## Materials and methods

### Research database

This retrospective cohort study collected data from 495 inpatients who underwent thyroid cancer surgery at our hospital between February 2019 and February 2021. The data was sourced from inpatient electronic medical records and other relevant records maintained at our hospital. A total of 268 patients were screened based on the inclusion and exclusion criteria. This study received approval from the Ethics Committee of Ganzhou People’s Hospital (ethics number: KY2019009341) and complied with the Declaration of Helsinki.

By analyzing the data provided in the patient’s medical records, the study subjects were divided into two groups based on their serum TSH levels 12 months after TC surgery. (1) TSH level conforming group, consisted of patients whose postoperative TSH suppression level was maintained at 0.5-2.0 mU/L; (2) Non-conforming group, consisted of patients whose postoperative TSH suppression level was either less than 0.5 mU/L or greater than 2 mU/L.

### Inclusion and exclusion criteria

Inclusion criteria: (1) Patients who are undergoing total or partial thyroid resection for the first time (and are considering) for indicated TC; (2) The postoperative pathological type of all patients is confirmed to be PTC. (3) After surgery, all patients are prescribed levothyroxine sodium tablets for TSH suppression therapy. (4) The clinical data for all patients are complete.

Exclusion criteria: (1) Final thyroid pathology confirmed that it was a malignant thyroid tumor except for papillary carcinoma and a history of other malignant tumors of the neck; (2) History of preoperative use of drugs affecting thyroid function (amiodarone, estrogen, glucocorticoid, non-steroidal anti-inflammatory drugs, furosemide, and antineoplastic drugs, etc.); (3) Postoperative complications such as infection, chyle, etc.; (4) The patient had undergone a second thyroid surgery due to the recurrence and metastasis of disease after the first thyroid surgery; (5) The lack of complete clinical data.

### Research methods

**Fig. 1:**
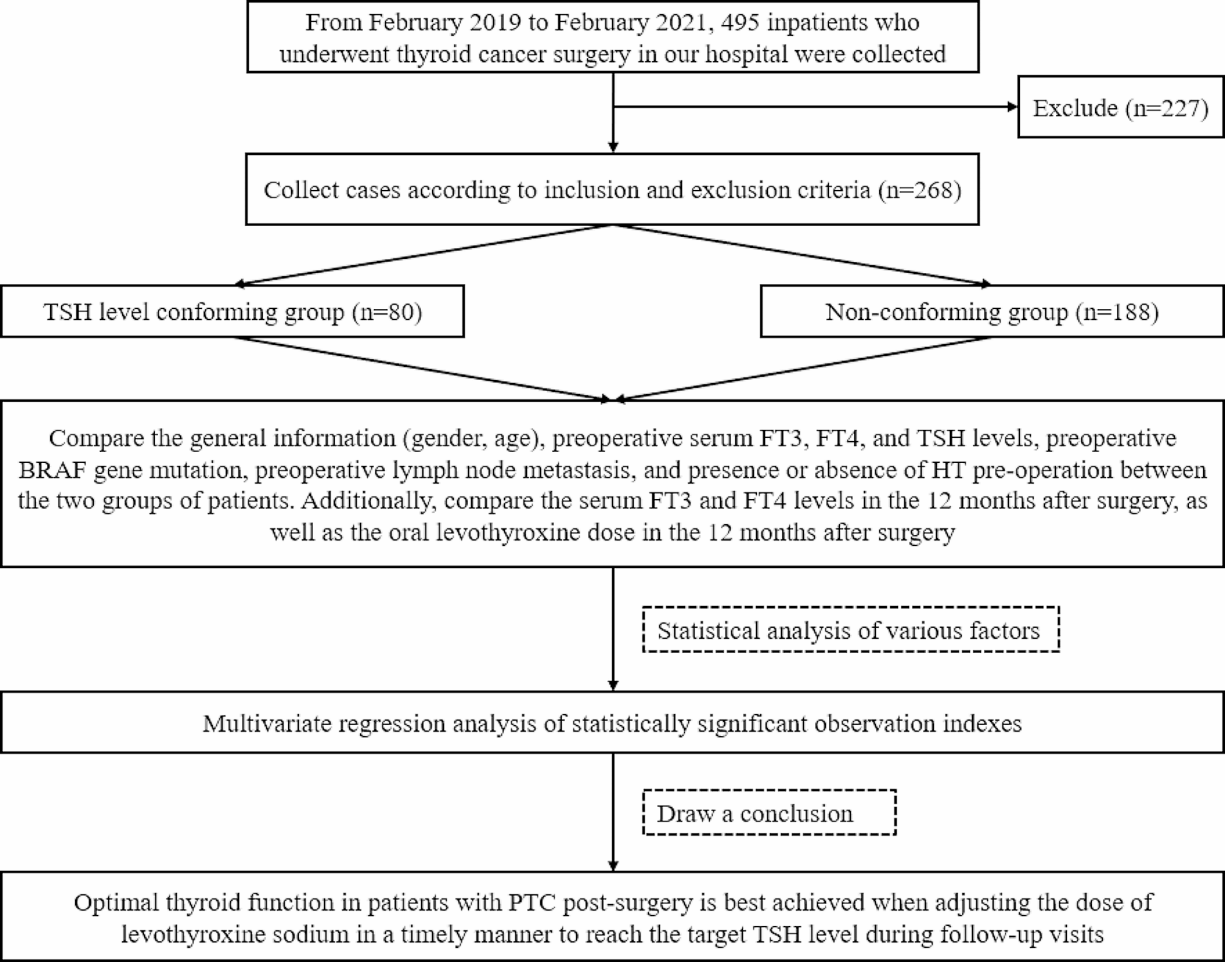
Flow chart for collecting case analysis and drawing conclusions

This study compared various factors between two groups, including general information (such as gender and age), preoperative serum-free triiodothyronine (FT_3_), serum-free thyroxine (FT_4_), serum TSH, the number of BRAF gene mutation cases, and lymph node metastasis. Additionally, the patient’s condition, tumor size, preoperative complications of Hashimoto’s thyroiditis (HT), postoperative FT_3_, FT_4_, oral levothyroxine sodium (Euthyrox) dosage, and other relevant information were considered. We conducted a further analysis to determine the correlation between these indicators and the success of TSH suppression therapy. Through this analysis, we identified the factors that influence TSH suppression therapy. The specific experimental flow is shown in Fig. 1.

### Collection of data

1. The study extracted general clinical information of the research subjects, such as patient name, gender, age, hospitalization number, weight, medication history, past medical history, surgical method, and chronic medical history.
2. The researchers collected preoperative and postoperative laboratory test indicators of the enrolled participants. The collected indicators encompassed serum levels of T3, T4, TSH, thyroperoxidase antibody (TPOAb), thyroglobulin antibody (TGAb), mutations in the BRAF gene, metastasis to lymph nodes, postoperative levels of FT_3_, FT_4_, TSH, TPOAb, TGAb, and the results of postoperative pathological examinations.
3. The patient’s preoperative and postoperative imaging examination results were obtained, which included preoperative thyroid color ultrasound results and postoperative follow-up thyroid color ultrasound results.

### Diagnosis of HT

1. The presence of elevated levels of TPOAb (> 35 IU/ml) or TGAb (> 116 IU/ml), along with or without diffuse thyroid enlargement, and with or without clinical and biochemical hypothyroidism.
2. If the postoperative pathological results indicate HT or lymphocytic thyroiditis, either of the two conditions can be considered HT.

### Determination of serum TSH level after operation

Standards for the diagnosis and treatment of TC provide guidelines for TSH suppression levels based on the risk group of the patients: (1) For high-risk patients, it is recommended to limit the initial TSH level to less than 0.1 mU/L; (2) For patients at moderate risk of recurrence, it is recommended to maintain initial postoperative TSH levels between 0.1 mU/L and 0.5 mU/L; (3) Risk stratification for patients with unmeasurable serum thyroglobulin (Tg) levels is low. TSH is restricted to a range of 0.5 mU/L to 2 mU/L, whether or not radioactive iodine therapy is administered; (4) Risk stratification for patients undergoing ^131^I ablation of thyroid tissue, and with low Tg levels, is determined by low levels of TSH suppression, specifically between 0.1 mU/L and 0.5 mU/L; (5) Those who do not undergo ^131^I ablation of thyroid tissue and have slightly higher Tg levels on follow-up, and are classified as low risk, it is recommended to inhibit TSH between 0.1 mU/L and 0.5 mU/L; (6) Typically, in patients who are undergoing thyroid lobectomy, the recommended target range for TSH suppression falls within 0.5 mU/L to 2.0 mU/L; (7) In cases where patients have unsatisfactory imaging results, it is recommended to indefinitely suppress TSH levels to less than 0.1 mU/L, unless there are contraindications; (8) To improve the effectiveness of serum treatment for patients, it is advised to regulate the medication within the range of 0.1 mU/L to 0.5 mU/L. This suggestion is grounded on the initial risk assessment conducted by the American Thyroid Association [18]. Additionally, it takes into account Tg levels, variations in Tg, and any unwanted responses connected to TSH suppression therapy; (9) For patients with an initial high recurrence risk stratification, but with a satisfactory treatment response (not evident clinically or serologically) or with uncertain efficacy, it is recommended to suppress TSH levels to 0.1 mU/L to 0.5 mU/L for a maximum of five years. Afterward, the degree of inhibition can be gradually reduced; (10) For patients whose treatment response is relatively satisfactory (not evident clinically or serologically) or whose efficacy is unclear particularly low-risk patients, it is recommended to maintain TSH suppression levels between 0.5 mU/L and 2.0 mU/L; (11) For patients who are satisfied with the curative effect of surgery alone or whose curative effect is unclear, no recurrence is observed on ultrasound, Tg levels are low or undetectable, Tg antibodies and TSH levels do not increase, and TSH is maintained between 0.5 mU/L and 2.0 mU/L [19]. The patients included in this study are all classified as low-risk patients. Based on the specified conditions (3), (6), (10), and (11), the target TSH level is set at 0.5-2.0 mU/L. The post-operative TSH suppression level is maintained within the range of 0.5-2.0 mU/L. If the TSH level falls within this reference range, it is considered to meet the standard; otherwise, if it is below or above this range, it is considered that the post-operative TSH suppression therapy level has not reached the standard.

### Statistical method

All statistical analyses were conducted using SPSS Version 20.0. The quantitative data in the TSH level conforming group and the non-conforming group were normally distributed and reported as mean ± standard deviation. Skewed distribution was presented as median (interquartile range). The means of the two groups with normal distribution and homogeneous variances were compared using t-test. When there was a non-normal distribution, comparisons between groups were performed using the rank sum test. Qualitative data were expressed as frequencies and percentages, and comparisons were made using the χ^2^ test. In this study, binary logistic regression analysis was employed to examine the impact of each observation index on postoperative TSH levels not reaching the target. The significance level was set at α = 0.05, and a *P* value < 0.05 indicated statistical significance.

## Results

### Comparison of general clinical data and clinical characteristics between the two groups

This retrospective cohort study focuses on patients who underwent thyroidectomy at Ganzhou People’s Hospital between February 2019 and February 2021. The research focuses on individuals who had their postoperative pathology report verifying PTC. Statistical analysis was conducted on a total of 268 patients who fulfilled the criteria for inclusion and exclusion. According to the group study on TSH suppression levels, the TSH level conforming group consisted of 80 patients, with 60 females (75.00%) and 20 males (25.00%). The average age of the TSH level conforming group was 47.51 ± 9.89 years. The non-conforming group consisted of 188 patients, with 148 female (78.72%) and 40 male cases (21.28%). The average age of the non-conforming group was 48.49 ± 10.17 years. The proportion of female patients was higher in both groups, and the age of onset ranged between 40 and 50 years old. Table 1 displays the general clinical data of the two patient groups, and there were no noteworthy variations observed that held statistical significance (*P* > 0.05).

Among the patients in the TSH level conforming group, 27 patients (33.75%) were found to have lymph node metastasis. Similarly, in the non-conforming group, 58 patients (30.85%) were observed to have lymph node metastasis. The median tumor size of patients in the TSH level conforming group was 1.1 (0.8, 1.5) cm, while the median tumor size of patients in the non-conforming group was 0.9 (0.6, 1.4) cm. In the TSH level conforming group, 66 patients (82.50%) had BRAF gene mutations, while in the non-conforming group, 146 patients (77.66%) had BRAF gene mutations. Table 1 illustrates that there exists no notable divergence in the clinical pathological features between the two groups of patients (*P* > 0.05).

**Table 1:** Comparison of general data and clinicopathological features between the two groups

General clinical data	Postoperative TSH suppression therapy		t/χ^2^	*P*
	TSH level conforming group (*n* = 80)	Non-conforming group (*n* = 188)		
Average age (years)	47.51 ± 9.89	48.49 ± 10.17	1.438	0.205
Gender (n, %)			0.447	0.503
Male	20 (25.00%)	40 (21.28%)		
Female	60 (75.00%)	148 (78.72%)		
Lymph node metastasis (n, %)	27 (33.75%)	58 (30.85%)	0.217	0.640
Tumor size (cm)	1.1 (0.8, 1.5)	0.9 (0.6, 1.4)	-0.390	0.710
BRAF gene mutations (n, %)	66 (82.50%)	146 (77.66%)	0.795	0.372

### Comparison of preoperative laboratory examination results between the two groups

The average preoperative FT_3_ level of patients in the TSH level conforming group was 3.04 ± 0.34 pg/ml, while the average preoperative FT_3_ level of patients in the non-conforming group was 2.99 ± 0.42 pg/ml. The average preoperative FT_4_ level of patients in the TSH level conforming group was 1.31 ± 0.22 ng/dl, while the average preoperative FT_4_ level of patients in the non-conforming group was 1.33 ± 0.20 ng/dl. There was no statistically significant difference in the average FT_3_ and FT_4_ levels between the two groups (*P* > 0.05).

There were 23 patients (28.75%) with preoperative TSH ≥ 2.0 mU/L in the TSH level conforming group and 112 patients (64.89%) with TSH ≥ 2.0 mU/L in the non-conforming group. There were 57 (71.25%) patients with preoperative TSH < 2.0 mU/L in the TSH level conforming group, and 76 patients (35.11%) with preoperative TSH < 2.0 mU/L in the non-conforming group. In Table 2, it is evident that the prevalence of patients with preoperative TSH ≥ 2.0 mU/L in the Non-conforming group was greater compared to the TSH level conforming group (*P* < 0.05).

**Table 2:** Comparison of preoperative laboratory examination results between the two groups

Data of laboratory examination before operation	Postoperative TSH suppression therapy		t/χ^2^	*P*
	TSH level conforming group (*n* = 80)	Non-conforming group (*n* = 188)		
Preoperative FT_3_ level (pg/ml)	3.04 ± 0.34	2.99 ± 0.42	0.698	0.467
Preoperative FT_4_ level (ng/dl)	1.31 ± 0.22	1.33 ± 0.20	-0.910	0.385
Preoperative TSH level				
TSH ≥ 2.0 mU/L	23 (28.75%)	112 (64.89%)	21.330	**< 0.001**
TSH < 2.0 mU/L	57 (71.25%)	76 (35.11%)		

### ROC curve analysis

**Fig. 2:**
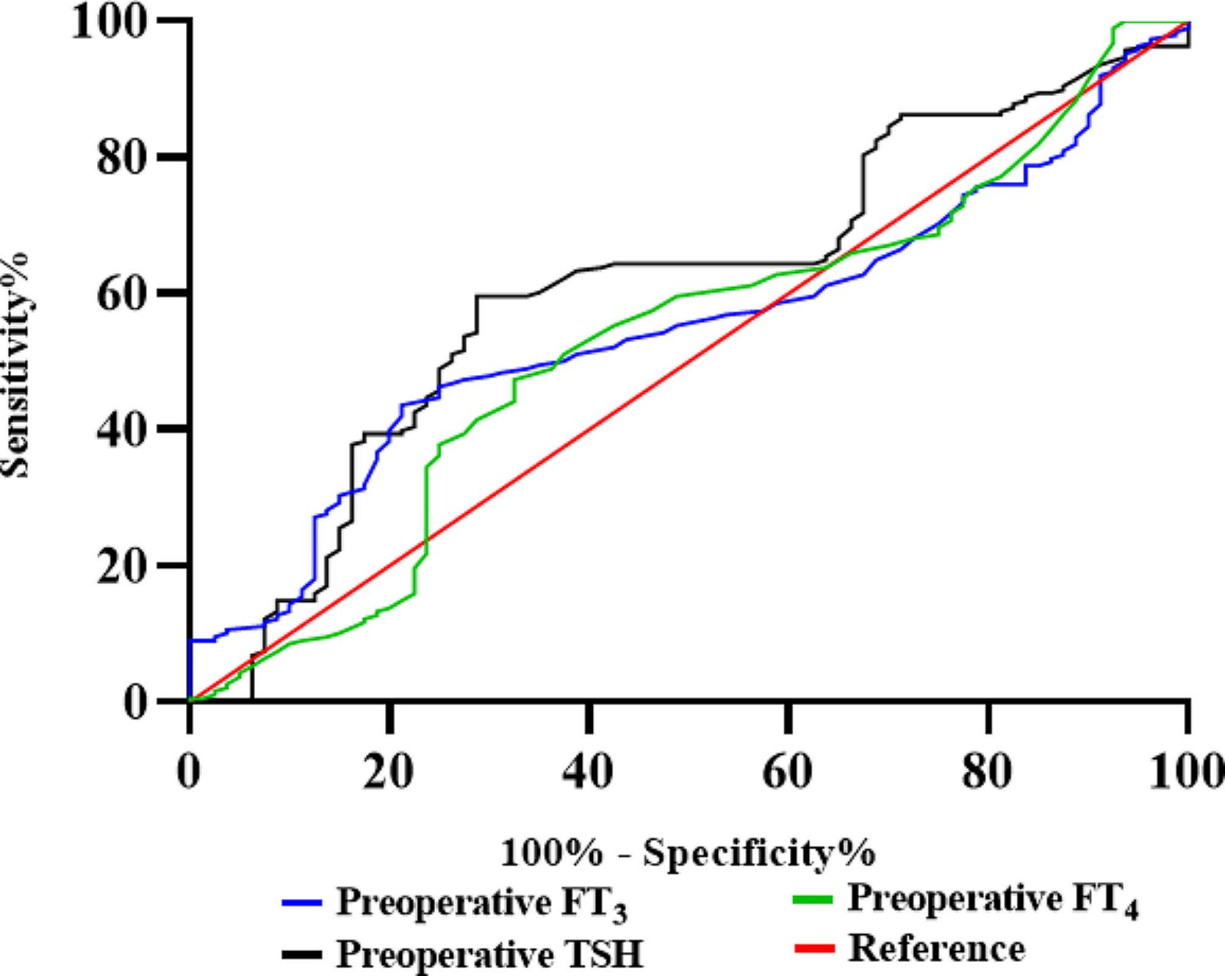
The accuracy of ROC curve analysis of preoperative FT_3_, FT_4,_ and TSH levels in predicting substandard prediction of patients with PTC after the operation

This study analyzes the accuracy of preoperative FT_3_, FT_4_, and TSH levels in predicting patients with PTC who do not reach the target after surgery. ROC curves were drawn for two groups of patients. The results indicate that the area under the curve for the preoperative FT_3_ indicator is 0.542 (*P* = 0.1962), the preoperative FT_4_ index is 0.528 (*P* = 0.4679), and for the preoperative TSH index is 0.610 (*P* = 0.0048). The difference in the area under the curve for the preoperative TSH index was found to be statistically significant (*P* < 0.05), as shown in Fig. 2.

### Comparison of two groups of patients with HT

During TSH suppression therapy, 24 patients (30.00%) in the TSH level conforming group were diagnosed with HT, while 56 patients (70.00%) did not have this complication. Among the patients in the non-conforming group, 105 patients (55.85%) had HT, while 83 patients (44.15%) did not have HT. Table 3 demonstrates that the incidence of HT in the non-conforming group was notably greater when undergoing postoperative TSH suppression therapy in contrast to the TSH level conforming group (*P* < 0.05), as indicated by the findings obtained.

**Table 3:** Comparison of two groups of patients with HT

With or without HT, *n* (%)	Postoperative TSH suppression therapy		χ^2^	*P*
	TSH level conforming group (*n* = 80)	Non-conforming group (*n* = 188)		
Yes	24 (30.00%)	105 (55.85%)	15.020	**< 0.001**
No	56 (70.00%)	83 (44.15%)		

### Comparison of oral dose of levothyroxine sodium tablets between the two groups after operation

In this study, 53 patients (66.25%) in the TSH level conforming group received postoperative TSH suppression therapy with levothyroxine sodium tablets at a dose of ≥ 1.4 µg/kg·d, while 27 patients (33.75%) received levothyroxine sodium tablets at a dose of < 1.4 µg/kg·d; Among the patients in the non-conforming group, 6 patients (3.19%) required a dose of ≥ 1.4 µg/kg·d of levothyroxine sodium tablets for postoperative TSH suppression therapy, while the remaining 182 patients (96.81%) required a dose of < 1.4 µg/kg·d. The rate of Postoperative TSH suppression therapy with oral levothyroxine sodium tablets at a dose of ≥ 1.4 µg/kg·d was higher in the TSH level conforming group compared to the non-conforming group (*P* < 0.05), as shown in Table 4.

**Table 4:** Comparison of oral dose of Euthyrox between the two groups after the operation

An oral dose of Euthyrox after the operation (µg/kg·d)	Postoperative TSH suppression therapy		χ^2^	*P*
	TSH level conforming group (*n* = 80)	Non-conforming group (*n* = 188)		
≥ 1.4	53 (66.25%)	6 (3.19%)	130.000	**< 0.001**
< 1.4	27 (33.75%)	182 (96.81%)		

### Comparison of follow-up frequency between two groups

In this study, among the patients who received postoperative TSH suppression therapy, 15 patients (21.13%) in the TSH level conforming group had a postoperative follow-up frequency of ≤ 2 times per year, while 56 patients (78.87%) had a follow-up frequency of more than 2 times per year; Among the patients in the non-conforming group, 58 patients (34.94%) had a postoperative follow-up frequency of 2 times or less per year, while 108 patients (65.06%) had a follow-up frequency of more than 2 times per year. The proportion of patients with a follow-up frequency of more than 2 times per year was higher in the TSH level conforming group than in the non-conforming group (*P* < 0.05), as shown in Table 5.

**Table 5:** Comparison of postoperative follow-up frequency between the two groups

Postoperative follow-up frequency (times/years)	Postoperative TSH suppression therapy		*χ* ^*2*^	*P*
	TSH level conforming group (*n* = 80)	Non-conforming group (*n* = 188)		
≤ 2	15 (21.13%)	58 (34.94%)	4.452	**0.035**
>2	56 (78.87%)	108 (65.06%)		

### Comparison of related indexes of thyroid function between the two groups

The average postoperative FT_3_ level in the TSH level conforming group after surgery was 2.97 ± 0.31 pg/ml, while the average FT_3_ level in the non-conforming group after surgery was 2.92 ± 0.64 pg/ml; The average postoperative FT_4_ level in the TSH level conforming group was 1.88 ± 0.32 ng/dl, while the average postoperative FT_4_ level in the non-conforming group was 1.73 ± 0.44 ng/dl. The postoperative FT_3_ and FT_4_ levels of patients in the TSH level conforming group were found to be higher than those in the non-conforming group (*P* < 0.05), as shown in Table 6.

**Table 6:** Effect of postoperative TSH suppression therapy on thyroid function

Thyroid function	Postoperative TSH suppression therapy		t	*P*
	TSH level conforming group (*n* = 80)	Non-conforming group (*n* = 188)		
Postoperative FT_3_ level (pg/ml)	2.97 ± 0.31	2.92 ± 0.64	2.335	**0.042**
Postoperative FT_4_ level (ng/dl)	1.88 ± 0.32	1.73 ± 0.44	4.187	**<0.001**

**Binary logistic regression analysis of influencing factors of substandard TSH level during postoperative TSH suppression therapy in patients with PTC**.

The results of the binary logistic regression analysis revealed that three factors, namely “preoperative TSH ≥ 2.0 mU/L”, “postoperative oral levothyroxine sodium tablet dose < 1.4 µg/kg·d”, and “combined with HT”, had positive regression coefficients and OR values greater than 1. This indicates that these three factors are associated with an increased risk of TSH levels not reaching the target after surgery (*P* < 0.05). The risk of not achieving the target TSH levels following suppressive therapy for PTC is amplified by 6.8, 75, and 2.8 times respectively. Among the influencing factors, there was no significant difference found after analysis for “follow-up frequency of ≤ 2 times per year” (*P* > 0.05), as shown in Table 7.

**Table 7:** Binary logistic regression analysis of the influencing factors of substandard TSH level after operation

	Regression coefficient (β)	Standard error (SE)	Wals	*P* value	OR value	95% CI
Postoperative TSH level ≥ 2 mU/L	1.941	0.489	15.433	**< 0.001**	6.875	2.640-18.207
Levothyroxine sodium tablet dose<1.4 µg/kg·d	4.342	0.595	54.098	**< 0.001**	75.173	23.844-244.652
Combined with HT	1.059	0.461	5.532	**0.020**	2.797	1.188–6.798
Follow-up frequency of ≤ 2 times per year	0.522	0.480	1.147	0.331	1.761	0.589–4.197
Constant	-3.836	0.662	33.568	**< 0.001**	0.024	

Note (Assignment): TSH level conforming = 0, TSH level non-conforming = 1; Levothyroxine sodium tablet dose<1.4 µg/kg·d = 1, Levothyroxine sodium tablet dose ≥ 1.4 µg/kg·d = 0, Combined with HT = 1, Without HT = 0; Follow-up frequency of ≤ 2 times per year = 1, Follow-up frequency of > 2 times per year = 0

## Discussion

DTC is predominantly characterized by PTC. Effective management of this prevalent form of DTC involves prioritizing surgical interventions. However, after surgery to remove most or all of the thyroid tissue, there is a decline in serum thyroid hormone levels, which leads to hypothyroidism and an inability to maintain normal hormone levels in the body. This decline in hormone levels fails to inhibit the pituitary gland from producing TSH due to feedback regulation. As a result, excessive amounts of TSH can induce the proliferation of cells in the thyroid gland (TC cells) and the synthesis of thyroglobulin. Therefore, TSH suppression therapy is another crucial treatment method for postoperative TC [20]. This study comprised 268 patients aged between 40 and 50 years with PTC who underwent TSH suppression therapy. A study was conducted to analyze the clinical data of TC patients, which included information such as patient gender, age, occupation, place of residence, ultrasound results, and pathological results. The findings revealed that the peak age of TC incidence in both men and women is between 41 and 50 years. Nevertheless, the rate of occurrence is considerably greater among females in comparison to males [21]. Within this investigation, both cohorts exhibited a larger proportion of female individuals diagnosed with PTC, and the onset age spanned from 40 to 50 years, corroborating findings from prior research. Thus, it can be concluded that gender and age do not affect the success of TSH suppression therapy after PTC surgery. Additionally, the age and gender distribution in both patient groups were similar and comparable. The cytoplasmic serine/threonine protein kinase encoded by the BRAF gene is regulated by its upstream RAS protein kinase. It performs functions related to cell proliferation, differentiation, and programmed cell death [22]. Research has indicated that the prevalence of the BRAF-V600E gene mutation is highest in PTC, compared to other types of TC. This mutation may be associated with the clinicopathological characteristics of PTC [23, 24]. The BRAF-V600E gene mutation can cause hypermethylation and silencing of the TSH receptor gene promoter, resulting in inadequate thyroxine production and increased TSH levels. It also accelerates internal stimulation of TSH and increases PTC genome instability and invasiveness [25]. No significant disparity in the mutation rate of the BRAF gene was identified between the two groups during this investigation. This could be because it only affects increased tumor invasiveness and surgical resection rate prior to treatment. There was no observed influence of the BRAF gene mutation on the efficacy of TSH suppression therapy following surgical intervention.

Suppressing thyrotropin is a crucial approach for treating patients with differentiated TC after surgery. To explore the connection between preoperative serum levels of TSH and thyroxine (T4) and the likelihood of postoperative recurrence of PTC, a retrospective analysis was conducted on data from 1578 PTC patients. The findings of this investigation demonstrated that elevated preoperative serum levels of TSH and FT_4_ were linked to an increased probability of PTC recurrence [26]. In a study conducted by scholars, a group of 168 patients with PTC were examined. The findings revealed that lower preoperative TSH levels were the most significant factor in predicting postoperative TSH levels below 2 mU/L [27]. According to the findings of this investigation, a larger number of patients were observed in the non-conforming category displaying preoperative TSH levels ≥ 2.0 mU/L. A substantial dissimilarity was detected between the two groups, which is consistent with prior research outcomes. It is postulated that individuals possessing preoperative TSH levels ≥ 2.0 mU/L face an increased possibility of failing to attain the designated criterion subsequent to undergoing surgery. To examine the correlation between the preoperative TSH level and the non-compliance rate of TSH suppression therapy, an additional evaluation was performed through binary logistic regression analysis. The results showed that the logistic regression coefficient was positive and the OR value was greater than 1, indicating a close association between preoperative TSH levels ≥ 2.0 mU/L and postoperative TSH levels. This increased risk of not reaching the target TSH levels is consistent with previous research findings. Patients with higher preoperative TSH levels require higher postoperative TSH suppression doses. If the oral dose cannot achieve this suppression dose, the compliance rate of TSH suppression therapy will decrease. Preoperative TSH levels ≥ 2.0 mU/L can increase the risk of suboptimal suppressive therapy. Therefore, in the clinical management of this patient group, the oral thyroid hormone dose needs to be appropriately increased to achieve a better suppressive effect.

HT is the most common form of autoimmune thyroiditis and can lead to hypothyroidism. Over the past decade, there has been an increase in its incidence in the population. Scholars conducted a meta-analysis to assess the influence of HT on the outlook of PTC. They detected 11 studies evaluating the prognosis of PTC, and two of these studies indicated that individuals with HT exhibited a superior overall prognosis in comparison to those lacking the condition [28]. The current investigation discovered that individuals diagnosed with PTC, but lacking HT, exhibited a heightened level of adherence to TSH suppression therapy following surgery, in comparison to those with HT. A notable dissimilarity between the two groups, as previously stated, was observed. The findings of the study remained consistent and, additionally, a binary logistic regression analysis was carried out to explore the correlation between the rate of non-compliance with TSH suppression therapy and HT. The results indicated a positive logistic regression coefficient and an OR value greater than 1, both of which were statistically significant. The rate of non-compliance with TSH suppressive therapy in patients with PTC after surgery increased by 2.8 times. When patients with PTC undergo surgery combined with HT, the remaining thyroid tissue is still exposed to HT autoantibodies. This exposure further exacerbates postoperative hypothyroidism and increases negative feedback. The increased secretion of high TSH leads to elevated blood TSH concentration and affects the effectiveness of postoperative TSH suppression therapy. We hypothesize that this pathological process contributes to the higher non-achievement rate observed in patients with HT.

Thyroid hormone replacement therapy is crucial for the postoperative care of patients with PTC. In a retrospective study involving 452 patients with differentiated TC, researchers discovered that only 4.9% of patients consistently used levothyroxine sodium tablets at a stable dose for four consecutive years. Additionally, approximately 50% of patients required treatment adjustments due to fluctuations in TSH levels. It was recommended to modify the dosage of levothyroxine sodium tablets during annual follow-up evaluations [29]. A binary logistic regression analysis was conducted to validate the potential risk of not achieving the target postoperative TSH levels when the dosage of oral levothyroxine sodium tablets is less than 1.4 µg/kg·d, thus further confirming the findings of this study that ≥ 1.4 µg/kg·d dosage enhances the compliance rate of postoperative TSH suppression therapy. The results revealed a positive logistic regression coefficient and an OR value greater than 1 (OR = 75.173), suggesting that the non-conforming group, with a dosage of levothyroxine sodium tablets less than 1.4 µg/kg·d, was associated with an increased risk of not reaching the target postoperative TSH levels. To prevent inadequate dosage and suboptimal suppression of TSH levels, it is important to adjust the dosage of levothyroxine sodium tablets in a timely manner during the postoperative follow-up of patients with PTC. To identify any remaining or recurring tumor formations, it is crucial to regularly monitor patients who have undergone either complete or partial removal of the thyroid gland for PTC. The frequency of these check-ups should be every six months to promptly identify any signs of tumor growth. Additionally, regular monitoring is necessary to assess the effectiveness of postoperative TSH suppression therapy. This study examined the follow-up frequency of patients who underwent TSH suppression therapy after surgery. The findings revealed that patients with a follow-up frequency greater than 2 times per year exhibited higher compliance rates compared to those with a follow-up frequency of 2 times or less per year. To further investigate the correlation between follow-up frequency and the achievement of postoperative TSH suppression therapy, a binary logistic regression analysis was conducted. Based on the findings, it was discovered that no substantial correlation existed between the two variables. This outcome can be ascribed to the notable frequency of follow-up among the participating subjects, wherein a significant portion of the patients had a follow-up frequency exceeding 2 times annually. Consequently, it does not significantly impact the overall standard of TSH suppression therapy. The lack of thyroid hormone production in patients with PTC after surgery leads to post-operative hypothyroidism, which results in endocrine disorders. Therefore, exogenous thyroid hormone is often administered to supplement thyroid function and maintain normal levels. Additionally, all patients in this study underwent long-term oral administration of levothyroxine sodium tablets for TSH suppression therapy. This not only effectively maintains thyroid-related hormones in the body but also helps in maintaining postoperative TSH suppression levels to prevent tumor recurrence. There was no significant disparity observed when comparing the preoperative levels of free triiodothyronine and free thyroxine between the two patient groups. Nevertheless, scrutinizing the postoperative levels disclosed no noteworthy distinction either. It was found that the average levels of FT_3_ and FT_4_ in the TSH level-adhering group were significantly greater than those in the non-standard group. This indicates that TSH suppression therapy demonstrates superior efficacy in preserving thyroid-associated hormone levels as compared to the group that fails to meet the desired target.

## Conclusion

Preoperative TSH levels ≥ 2.0 mU/L, postoperative levothyroxine sodium tablet dosage < 1.4 µg/kg·d, and the presence of HT are associated with an increased risk of postoperative TSH levels not reaching the target. Appropriate dose of levothyroxine sodium for postoperative TSH suppression therapy in PTC needs to be determined by regular monitoring of individual patients to avoid suboptimal TSH suppression due to low dose of levothyroxine sodium and to achieve better thyroid function.

## Data Availability

The datasets generated and/or analysed during the current study are not publicly available due to individual privacy but are available from the corresponding author upon reasonable request.

## Abbreviations

ATC: Anaplastic thyroid cancer
DTC: Differentiated thyroid cancer
FT3: Free Triiodothyronine
FT4: Free Thyroxine
FTC: Follicular thyroid carcinoma
HT: Hashimoto thyroiditis
MTC: Medullary thyroid cancer
PTC: Papillary thyroid carcinoma
T3: Triiodothyronine
T4: Thyroxine
TC: Thyroid carcinoma
TSH: Thyroid Stimulating Hormone
TST: Thyrotropin suppression therapy
Tg: Thyroglobulin
TI-RADS: Thyroid imaging reporting and data system for ultrasonography
TPO: Thyroid peroxidase
TPOAb: Thyroperoxidase antibody
TGAb: Thyroglobulin antibody
